# Rediscovering the Relative Deprivation and Crime Debate: Tracking its Fortunes from Left Realism to the Precariat

**DOI:** 10.1007/s10612-021-09554-4

**Published:** 2021-03-09

**Authors:** Craig Webber

**Affiliations:** University of Southampton, Southampton, UK

## Abstract

This article revisits the concept of relative deprivation and asks whether it is still useful for criminology. The article traces the way relative deprivation has been used in the past to understand crime and how it has connections to other, more recent, additions to debates on social justice. I argue that relative deprivation has disappeared even in the place that it had become the key explanation for crime—left realism. In so doing, I explore the resurrection of left realism in criminology—what I refer to as “post-millennial left realism”—first, by those who were associated with it originally, and then with Hall and Winlow’s (2015, 2017) shift in emphasis to what they term “ultra-realism.” I maintain that relative deprivation is still a powerful concept for bridging several related areas that should still be central to the concerns of criminology—in part, because it is still a major concern in popular social science and social psychology. Why has it disappeared in criminology? I present an argument that suggests that the absence of certain research methods, such as ethnographic and qualitative or small-scale survey methods, has impoverished our understanding of the lived reality of people experiencing the social transformations of a networked, precarious society. The massive polarization and disruption in politics and social discourse, as well as the worldwide economic, public health, and social transformations (ranging from the #MeToo and Black Lives Matter protests to the COVID-19 global pandemic) have demonstrated the continued relevance and analytical power that relative deprivation, in its elaborated form, brings to questions of crime and justice.

## Introduction

One of the fullest accounts of the concept of relative deprivation was found in the work of W. G. Runciman in 1966. Since then, the concept of relative deprivation—the idea that people may feel deprived on any dimension in comparison to others in society—has seen its status as a theory that might explain crime rise and fall. Criminologists that collected under the moniker of “left realism” originally identified relative deprivation as a central cause of crime. Relative deprivation has been largely absent from criminology for many years, however, despite the re-emergence of realist criminology in various forms. This article represents a sequel, of sorts, to an article I wrote several years ago (Webber 2007a).

The reason for re-visiting the concept now is that the original arguments for its re-evaluation are still relevant in a world that has changed dramatically since my earlier work (see, e.g., Webber and Yip 2018 for an application of the concept to “hacktivism”). I argue that the analytical worth inherent in the concept is more pertinent now than ever before. Indeed, in 2008, just after writing the first article, the world economy crashed and ushered in a politics of austerity. Since then, radical changes in employment have created the “gig economy” (independent contractors paid by the project or task), zero-hour contracts (which allow employers to offer work in a piece-meal way, and that the worker can refuse to work if they^1^ do not need to) and automation through artificial intelligence (AI), robotics and drones. “Big data” has appeared with all the zeal that surrounded previous positivistic accounts of human nature. The difference is that these data have become monetized by the colossal power of the big technology companies. Social media, navigation services, search engines, smart phones and speakers, and doorbells with cameras linked to the web produce real-time pictures of anyone using them. Despite the commodification of our data by technology companies, there has also been one of the biggest shifts in the power of the public to target the wealthy and powerful, energized by the influence and reach of social media—and evidenced by the #MeToo and Black Lives Matter movements (Webber and Yip 2018). In this, we have seen the way that protest oscillates between the online and the offline, from the digital realm to the embodied and vulnerable locations of the street, social spaces and homes. We have moved with dizzying speed from the first Black President of the United States (US), Barack Obama, to Donald J. Trump, who used the military to disperse crowds peacefully protesting the extra-judicial killing of a Black man, George Floyd, in Minneapolis in May 2020. Throughout 2020 and into 2021, COVID-19 has caused millions of deaths, a worldwide economic crash and the need for collective sacrifice on a global scale. This has resulted in a struggle to create a narrative from all the competing threads of a story. The rise of “fake news” and propaganda of phenomenal reach has fought against what the early web pioneers thought would be the spread of democracy from the ground up (Wu 2010). Throughout all of this, we have witnessed the epoch-shattering creation, dissemination and deeply embedded network of computers into the World Wide Web. Against this backdrop, I argue that relative deprivation is a key explanatory variable.

Consider that in a critical review of David Garland’s *Culture of Control* (2001), one of the pioneers of left realism, Jock Young, argued against Garland’s contention that criminology was moving away from theories of relative deprivation and toward greater reliance on control theories. Young maintained that the evidence pointed to a different shift—one *toward* theories of relative deprivation as the motivating cause of crime in criminological research and public explanations of crime in surveys (Young 2003b). While Young may have been right then—at the end of the 1990s and early 2000s—it is not the case now, although outside of criminology, in political theory, psychology and social policy, the concept of relative deprivation is still employed and it is still being developed. This article reflects on the absence of relative deprivation in criminological scholarship and makes some tentative suggestions and justifications for its recovery.

Briefly, relative deprivation can be defined as the excess of expectations over the opportunities for attaining them (Webber 2007a). This article will trace the use and neglect of relative deprivation through left realism, which I will split into a pre- and post-millennial tradition. From here, I move to the new ultra-realism, the precariat debate, and social psychology. Such an exploration requires breadth at the expense of depth. I argue that research into relative deprivation and social justice should be central to criminology—and essential to a critical criminology with aims to alter not just the harms *of* crime, but the harms that *lead to* crime. More than that, one of the main lessons that needs to be learned by the recent political events that are often presented as shocks, from the Arab Spring, the climate crisis, to Brexit, Trump, #MeToo, Black Lives Matter and COVID-19, is that there is a need for a return to a subjective epistemology. Big data and algorithms based upon it, election polling, and social scientific research have all been complicit in producing positivistic accounts that flatter to convince, and are then embarrassed by the speed of their reversal (see, e.g., Young 2011). This is just as true for criminology as any other discipline. Relative deprivation occurs when there is a negative mismatch or contradiction between empirical “reality” and “real” life. Consequently, this article concludes by showing that relative deprivation, with its dual focus on the interaction between the subjective and the objective, is a necessary analytical device for interrogating the how and the why of these “shocks.” I argue that relative deprivation is a crucial analytical tool to help in the understanding of how the world is rendered objectively, through statistics, election polling and data trails. I then scrutinize these analyses using a qualitative or ethnographic imagination. By doing so, we can ask fundamental questions about the relationships between objective, empirical accounts of the world, and the lived reality of those who experience the world *as it is*.

## W. G. Runciman's Relative Deprivation: The Missing Link between Merton and Pre-Millennial Left Realism

Relative deprivation was a concept central to the left realist tradition of criminology (Lea and Young 1993/1984). It was, and still is, founded on the idea that anyone can feel deprived of something irrespective of the person’s place in the social hierarchy. Therefore, relative deprivation extends further than the idea of *absolute* deprivation, which posits that only those at the bottom of the social structure suffer the most. In left realist criminology, relative *economic* deprivation represented the central cause of crime because it could explain crime committed by anyone—rich or poor. Moreover, relative deprivation could account for any crime, from theft to violence.

Relative deprivation owes much to Robert K. Merton’s ideas about social structure and anomie in the US in the mid-1930s, and the subsequent form those ideas took in strain theory (Merton 1938; see also Agnew 1992, 1999, 2006; Agnew et al. 1996; Lea 1992; Messner and Rosenfeld 2012; Young 1992, 2003a). To risk simplifying an often-misunderstood theory, I would stress that Merton argued that the main goals in society are success, wealth and a conventional family unit—the so-called “American Dream.” These goals were not available to everyone equally, however. Some individuals and groups felt strain by being denied access to the legitimate routes to achieve material success, such as poor education, poor health and a lack of adequate housing.

Merton had two doctoral students who extended his original work. Albert Cohen (1955) focused on non-acquisitive behavior, such as vandalism, in order to show how strain can result in crime that does not have material value. Using the Freudian notion of reaction-formation, Cohen showed how young boys symbolically attacked that which they desired. Merton’s other student, Richard Cloward, who with Lloyd Ohlin, took Merton’s original ideas about strain and added an element of opportunity (see Cloward and Ohlin 1960). Not everyone knew the right people or had the right skills to be able to engage in all sorts of crime. Many young people were, therefore, alienated from both the legitimate opportunity structure and the illegitimate opportunity structure. Put simply, not everyone knew how to transform the loot from a burglary into money; only some could steal a car, to offer another example.

Nevertheless, further theoretical elaboration of relative deprivation did not take place within left realism, where the concept served more as a descriptive device to explain the “aetiological crisis.” This was a term that Jock Young used to describe how crime rates increased as affluence decreased and vice versa, allowing Young to argue that the “poverty causes crime thesis” was fundamentally flawed. As such, despite the left realists arguing that the theory could explain all types of crime, its earliest application tended to be limited to volume crime (the majority of all crimes and so those with the greatest impact on victims, e.g., assault, burglary, theft, vandalism) that affected the poor and working class. Central to relative deprivation is the idea of subjectivity—that people often make judgments that are contradictory to their own best interests and that rationality is bounded by error (van Hardeveld et al. 2017). Relative deprivation serves as an empirically grounded critique of rational choice theory (Clarke and Cornish 1985; Kahneman and Tversky 1979), and this is the narrative that runs throughout this review of the concept. As noted above, relative deprivation, as conceptualized by the left realists, derives from Merton’s notion of anomie, but it has been more fully and explicitly outlined in *The American Soldier* (1949) by Stouffer and colleagues and subsequently in *Why Men Rebel* (1970) by T.R. Gurr. Its most complete elaboration appears in *Relative Deprivation and Social Justice* (1966) by Runciman. Runciman’s account of the theory is the one that has had more influence on later research, especially in the field of social psychology.

One of the most significant aspects of relative deprivation theory is the way it can supersede the polarized debate between the left and right on the political and academic spectrum. This debate about the link between the cause of crime and social class followed two distinct and diametrically opposed paths. Marxist and other leftist social scientists emphasized poverty as a motivation for crime and the agents of capitalism as responsible for criminalizing the (activities of the) poor, at the same time that those agents ignored or legitimized crime and deviance committed by the powerful (Hall et al. 2013/1978; Taylor et al. 1973; see also Currie 2019). Those on the right, on the other hand, focused on individual-level factors, such as the person’s volition or biological and psychological impairments, rather than on the social structure of capitalist societies (Herrnstein and Murray 1994; Wilson 1983). The representation of class in both of these debates was, and continues to be, primarily one of involving the imposition of pre-existing definitions and metrics. Effectively, those defined as the poor, powerless, precariat or working class tend to have this status imposed on them by politicians, journalists and criminologists (among others). Moreover, rarely did these early accounts take into consideration the different experiences of, for example, women or ethnic minorities. In addition, politics tends to be “done to them” in that the poor are either *de facto* or *de jure* disenfranchised. Runciman’s approach differed, however, in so far as it focused on *self-defined* class position, meaning that comparisons could be made between individual’s class position with which they self-identified and that which would be imposed when reference was made to economic indicators and occupations, such as in a census. Runciman’s method enabled actors to subjectively place themselves within a stratified hierarchy—arguably a more accurate reflection of how people perceive themselves. This aspect of the concept of relative deprivation, when it was used in left realism, allowed for the perspective to become a political project of reform. Using local victim surveys (in contrast to the state-originated national crime surveys, such as the British Crime Survey or National Crime Victimization Survey in the US), left realists were able to show that the media and political narratives of crime in inner-city housing estates did not match the perception of those that lived there (Jones et al. 1986). The picture painted by these official and media accounts was of communities riven by crime and unwilling to help the authorities to rectify the problem. The local victim surveys showed this to be falsely constructed (see also MacLeod (1995/1987) and Nightingale (1995) for ethnographic accounts that provide similar accounts in the US at the same time). Communities wanted help, but the solution was a multi-faceted intervention involving democratic policing that reflected the needs of the communities and investment in housing, jobs and training. The absence of these basic services and amenities were merely aspirations for these communities. But these sorts of social investment were expectations in other communities. This was the root of the sense of relative deprivation and was, for the pre-millennial left realists, the root cause of crime.

Although Runciman’s work is largely missing in all forms of left realism, despite relative deprivation appearing throughout the pre-millennial form of left realism (Webber 2007a), we can see his influence in the arguments expressed above. For example, Runciman (1966: 3–4) set out to answer two related questions: “what is the relation between institutionalised inequalities and the awareness or resentment of them?” and “which, if any, of these inequalities ought to be perceived and resented—whether they are or not—by the standards of social justice?” Runciman was concerned with identifying the circumstances that led to feelings of resentment and highlighted three main sources of relative deprivation: class position, education and power. Frustration within one category does not necessarily mean frustration in the other two categories, which is different from Merton’s (1938) opportunity theory. For Merton, the American Dream was the key source of frustration. Runciman (1966: 10–11), however, argued that “[r]elative deprivation should always be understood to mean a *sense* of deprivation; a person who is ‘relatively deprived’ need not be ‘objectively’ deprived in the more usual sense that he is demonstrably lacking something” (emphasis in original).

The first aspect of Runciman’s perspective that needs to be addressed is the distinction between *relative* and *absolute* deprivation. To be “relatively deprived,” a person need not be suffering deprivations that are harmful to that person’s existence, as is the case in many definitions of absolute deprivation. Thus, relative deprivation applies to those who are poor and those who are rich and everyone in between. For example, Person A earning the average income for their country might compare themselves to Person B earning the same salary but feel deprived because Person B seems better able to invest money in a house that appreciates in value more than does Person A’s home. In other words, Person A has clothing, food, shelter and a stable job, but still feels relatively deprived when compared to Person B. But, crucially, this is only relative deprivation if a third party would deem this disparity as *unfair*, rather than as *envy*. As noted below, it has to be value-neutral. The sense of deprivation must be “earned” as a result of a failure of social justice. In this example, the feeling of deprivation would constitute relative deprivation if it could be established that certain areas had seen their house prices inflated unfairly through differential investment in the local economy.

The comparison to someone similarly situated is important because the theory predicts that we compare ourselves only rarely to others who are far removed from our socioeconomic position. For example, a homeless person tends not to compare themselves to a billionaire fund manager and feel deprived, but might compare themselves to another homeless person who has recently secured a rental property. Person A’s feelings or the first homeless person’s sentiments are subjective; they do not require accurate data for comparisons to occur. As I will argue, however, the technologically interconnected world has rendered the difference between groups *less* stark on some consumer variables, as evidenced by, for example, the ubiquity of televisions, cars or even smart phones. At the same time, though, this technologically interconnected world has widened political divides and broadened the chasms between political ideologies (such as ardent supporters of Donald J. Trump and Never-Trumpers or Brexiters versus Remainers). Indeed, I contend, creating relatively deprived in-groups who compare themselves unfavorably to out-groups is the driving force of politics and consumerism in the post-millennial world. The power to manipulate subjective measures of comparability for political and commercial gain, through “fake news,” “deep fakes” (digitally manipulating video using computer-generated images so that a person is made to look as if saying or doing something that they were not) and data-fed advertising, where companies harvest your personal data to personalize their marketing and messages, has increased exponentially as the internet has become embedded and ubiquitous (see generally Brisman 2018).

The second main element of Runciman’s approach is the idea that we measure ourselves against other comparative reference points, as in the example of the homeless individual above. This can occur at the individual level, so that one person compares themselves to another person and evaluates and subsequently determines themselves to be deprived in one or more dimensions, such as happiness, health or wealth. Runciman refers to this as *egoistic relative deprivation*. For Runciman, *fraternalistic relative deprivation*, in contrast, occurs when someone identifies with a particular group and feels relatively deprived as a member of that group in comparison to another group (for a discussion, see Taylor 2002). The fundamental question for Runciman is *when* do we make comparisons? Because relative deprivation is a subjective, rather than objective, sense of deprivation, one of the compelling aspects of the underlying comparative reference group theory is identifying those who do not feel deprived, but possibly should, and those who we might not expect to feel deprived, but do.

Runciman recognized that these questions served little purpose unless they could provide the driving force for a positive social policy outcome. To offer a mechanism for doing so, Runciman considered Rawls’ (1958) notion of “justice as fairness.” It is in this aspect of the concept that the subjective side of relative deprivation meets a more objective element where a sense of deprivation should be value-free. An observer should determine if a practice is fair even if they would be the loser rather than the gainer from the outcome.

An example would be a fair taxation system where everyone would pay their share even if that share was a higher proportion of their income than a poorer tax payer because they had accrued a relatively larger proportion of disposable income and were able to afford all basic needs even after paying the tax. An outsider should view the financial deprivation as fairly experienced and would come to the same conclusion without knowing whether or not they would have to pay more themselves. If it is determined that the tax system is unfair and the rich get away with paying a smaller amount of their incomes on tax, then an argument can be made to change the situation to reduce the unfair deprivation.

Of course, the key problem, here, is that there is an inherent liberal fantasy that suggests that people in advanced democracies sit and discuss fairness in the manner Rawls suggests. But that is precisely *why* it is such a useful idea when applied to criminological issues of what might be a factor in the tendency toward crime. Social justice is not fair; some groups do become marginalized from the fruits of an economy, the fairness of justice, and the stability of a job. The following parts look at the reinvigoration of realism in criminology to see if relative deprivation is still employed.

## Post-Millennial Left Realism: Relative Deprivation Retreats into the Shadows

Except for a few sporadic publications (e.g., DeKeseredy 2003; DeKeseredy et al. 2006), left realism all but disappeared into the shadows of textbooks and lecture theaters for criminology students in the late 1990s and early 2000s. One of the key pre-millennial left realists, the late-Roger Matthews, returned to relative deprivation briefly in his book, *Doing Time: An Introduction to the Sociology of Punishment* (2009b), and the discussion reflected a left realist form of argument. Since 2010, there has been a revival of a version of left realism. In a special edition of the journal *Crime, Law and Social Change* in 2010, the guest editors, Martin D. Schwartz and Walter S. DeKeseredy, argued that left realism was interested in the “here and now” because that is when the poor and disadvantaged are harmed. Somewhat surprisingly, of the eight articles comprising this special issue, only three referred to relative deprivation (Dragiewicz 2010; Gibbs 2010; Schwartz and DeKeseredy 2010). Moreover, John Lea, a key contributor to left realism, avoided mentioning relative deprivation in his more recent work (e.g., 2002, 2010, 2016). Indeed, none of these discussions used the idea of relative deprivation in anything more than a brief description of the earlier 1980s and 1990s version of left realism as it derived from Merton, American subcultural theory and strain theory (see also Currie 2010; DeKeseredy and Schwartz 2013; Jacobson and Chancer 2010).

In 2016, another special issue on left realism was published. As before, the similarity of post-millennial left realism to the pre-millennial version of left realism was partial, at best, and with only passing reference to relative deprivation (see, e.g., DeKeseredy 2016; Donnermeyer 2016; Hogg 2016; Renzetti 2016). The momentum to reinvigorate left realism continued in 2016 with the publication of an edited volume by Roger Matthews (2016b). These books and special issues of journals were missing some of the key analytical devices of pre-millennial left realism, and notably relative deprivation was almost entirely missing (see also Matthews 2010, 2014a, b).

### Shifting Further from Left Realism: Criminology’s Failure to Predict and Explain the Crime Drop

In another 2016 publication, Matthews (2016a) discussed the global drop in crime rates and dismissed the pre-millennial left realist position on deprivation and strain theory. Prior to Matthews’ (2016a, b) work, Farrell (2013) had outlined the key factors that had been put forward to explain the crime drop—from better security of devices and cheaper consumer durables to a rise in jobs and affluence to lead-free petrol and paint. Missing from the list of Farrell’s hypotheses, however, is anything remotely approaching a theory of the political economy of crime consistent with the previous approach of left realism, which had proposed relative economic deprivation as a cause of crime argument (see, e.g., Lea and Young 1993/1984; Young 1992). Like Farrell, Matthews (2016a; see also 2009a) also rejected deprivation as one of the principle causes posited for the rise in crime in left realism even as he revisited and attempted to reinvigorate the perspective. Indeed, what is peculiar is that as we see a rise in explanations that suggest a primary causal role for the political economy in various social transformations, from the gig economy to social relations unfolding on social media (Halford and Savage 2017; Piketty 2014; Putnam 2000; Standing 2011), so it might be expected that we see the same political economy arguments, including relative deprivation, reappearing to explain the crime drop in the reappearance of left realism. But this is not the case. As Matthews (2016a: 8) contends,

Standard liberal theories that attempt to explain the crime drop in terms of poverty, deprivation, unemployment or economic fluctuations have proved inadequate. One of the significant features of the crime drop is that it appears to transcend economic changes including changes in the level of unemployment. Standard versions of strain theory appear less than helpful in this respect and, despite the increasing gap between rich and poor in many advanced countries, recorded crime has decreased.

DeKeseredy (2016) maintains that maybe a return to the type of small-scale victim surveys that were the methodological driving force of early left realism might show the crime drop to be overly exaggerated (see also Jones et al. 1986; Winlow and Hall 2019). One might add other more ethnographic forms of research that might highlight the types of harm that more formal methods render opaque. Indeed, one could put forth a hypothesis that suggests that crime has dropped—at least, crime as counted through formal research methods of surveys and criminal justice statistics. But the harms caused by structural relative deprivation (e.g., widening disparity between rich and poor, precarious work futures, intersectional inequality) has manifested in the popular, and unpredicted, protests of the Arab Spring, #MeToo, Black Lives Matter, and the myriad democratic outcomes that bucked the predictions of psephologists and political commentators (e.g., Brexit and the election of Donald J. Trump). Relative deprivation does not always lead to crime.

Because the key concepts that once made up left realism are only partially relevant in the current form of left realism, what makes the recent work warrant the name, “left realism,” and does it matter? What if relative deprivation has not made the transition into the more recent forms of left realist thinking? Relative deprivation was always a thinly elaborated concept, as Matthews himself maintained (2016b). Relative deprivation also tended to be associated more with the work of Jock Young (1999, 2007, 2011) than any of the other academics involved in left realism. Citing Young’s *The Exclusive Society,* Hayward (2004) notes that the gaze of relative deprivation used to be to look to those who were better off, rather than the current position of looking down or across to those who are worse off but undeserving—the welfare scrounger or immigrant, as described by Young. The idea that relative deprivation *was* mainly about a “gaze upward” (Young 1999: 9; see also Sozzo and Fonseca 2016) ignores the very dynamic approach taken by Runciman and those, mainly social psychological accounts, that expanded Runciman’s work to show the multitude of different directions that the “gaze” might take, and at different levels of abstraction, be that a person, or group, or nation. So, to answer the question above—what if relative deprivation has not made the transition into the more recent forms of left realist thinking?—one might respond that relative deprivation was never used in pre-millennial left realism to the fullest of its analytical possibilities, and the fact that it is far less prominent in the post-millennial version might not matter at all if the questions to which relative deprivation might be employed to help find the answer were not still, or even more, relevant now. I will argue, however, that relative deprivation in its more developed form, as employed in political science and social psychology, is able to provide insight into how consumerism has become a key focus of work on the links between crime and culture that came after pre-millennial left realism.

## Crime, Consumerism and the Absence of Relative Deprivation in the New Ultra-Realism

Where earlier forms of left criminology tended to ignore or diminish the seriousness of crimes of the powerless (Currie 2019), the new ultra-realists (e.g., Hall et al. 2008; Raymen 2017; Treadwell 2013; Treadwell et al. 2013; Winlow 2014; Winlow and Hall 2016, 2019; Winlow et al. 2015) do not just accept that crime is a devastating blight on poor and powerless communities. Instead, they contend that we should widen our vision to many more forms of harm than are typically considered by criminology. In this, there is some overlap with left realism, where crime is a real and present problem, not a social construction to be ignored. This, however, is where ultra-realism’s overlap with left realism begins to falter. Hall and Winlow (2015: 62) take an unappreciative view of left realists and the original use of relative deprivation, claiming they were, “little more than ‘edgy administrators’, with a left-pragmatic approach that flattered to deceive, rather than critical realists determined to identify and grapple intellectually with capitalism’s structural forces and processes and their very real consequences.”

Ultra-realists argue that humans do not have an innate sense of justice—or an inbuilt universal sense of right or wrong—that relative deprivation can “work on,” thus rendering the theory invalid (Hall et al. 2008). If humans do not have a naturally occurring level of justice, then it cannot be impacted by being asked to consider if something is fair along the lines suggested by Rawls. Ultra-realists maintain that traditional, pre-millennial left realist criminology did not question the pursuit of status and power that comes from the acquisition of consumer durables, property and wealth (see also Agnew 2006). Ultra-realists contend, then, that left realism, both pre- and post-millennium, regard the American Dream as a natural and normal economic goal. Illegal deviations from the route to the goal can be held in check by social control mechanisms. In contrast, Hall and Winlow (2015), drawing on the work of Žižek (2005, 2014), assert that humans are not narcissistic nihilists chasing consumerism through some natural inclination to this condition. Instead, capitalism has created the urge to satisfy demands for consumption of products and services. The less well-off and powerless do not replace a consumerist value system for a resistant and confrontational system due to relative deprivation; instead, they embrace the consumerist system from which they are, in reality, excluded (Winlow and Hall 2013). The fix for this current predicament is to change the belief system so that competitive consumerism is replaced with another belief system.

Ultra-realism stands in contrast to early forms of radical criminology that preceded left realism and that was epitomized by *The New Criminology* (Taylor et al. 1973) and *Resistance Through Ritual* (Hall and Jefferson 1976)*.* Ultra-realists do this by noting that the poor, powerless and working class embrace consumerism and that there is lack of resistance to oppression. The poor and powerless *would be* hyper-consumers *if* they had the wealth to afford their desires. We see this in the conspicuous wealth being paraded in music videos, song lyrics, and the type of clothing that is purchased when funds allow (Winlow 2013). In Hall and Winlow’s view (2015), the harm of consumerism results in social inequality.

For example, the desire for cheap transportation inspires companies like Uber*.* Their business model is based on the ability to pay drivers different rates at different times of day—and sometimes not at all. Uber is a prime example of the precarious gig economy. This results in social inequality, but one that is seemingly chosen by the Uber driver. This is not an example of a top-down, capitalist exploitation of the poor and powerless, which was the subject of earlier Marxist-inspired forms of critical criminology. Rather, this is a model of complicit exploitation built into a business model that is presented as one that benefits everyone, especially those who need a few short hours’ work a week. Winlow and Hall (2013) contend that class identity has become subsumed by the quest for subjective identity—the individual search for self-actualization. In other words, Winlow and Hall (2013) maintain, class identification no longer drives politics; we are drawn to the surface level of representation, where identity becomes individualized at the expense of the collective. “The narrative of nation” replaces this lost collective class identity, and promotes neoliberal policies of small regulation—or “austerity” to use its more familiar label—as the patriotic way forward (Winlow and Hall 2013: 16).

Certainly, class, as it was once understood, might be less important now. But the forms of collective identity now are just as real and demonstrably influential as a fulcrum for social change. For example, the varied forms of collective protest, such as the Black Lives Matter protests against police brutality and racially motivated violence in the US in 2020 toward BAME (“Black and Minority Ethnic”) citizens, ignited both social media on the web and passions in the streets of cities around the world. The power of the web to publicize abuse of power—and then to act as the means to galvanize a collective, group response through fostering shared symbols of in-group solidarity, coupled with the frictionless communication afforded by networked-mobile devices—provided the means for the expression of collective anger and frustration. This is relative deprivation, supercharged by the web and resulting in mass civil protest in major cities around the world. For the pre-millennial left realists, relative deprivation caused crime. In the post-millennial world, relative deprivation has returned to being the more analytically powerful tool that can help explain not just crime, but also mass civil protest (Gurr 1970; Runciman 1966).

So, within the new ultra-realist reading of social economy and politics, what is the relationship to crime? The key question that ultra-realists try to emphasize is: “why do some individuals and groups risk harm to others as they pursue their instrumental and expressive interests?” (Hall and Winlow 2017: 401). In answering this question, they seek to distance themselves from social constructionism—the sociological position that suggests that every social institution is produced socially and is not a natural state of affairs (for example, capitalism is *created*, rather than *preordained*). Similarly, new ultra-realists criticize the identity politics of intersectionality—the study of how different social variables such as class, gender, race and sex can combine to produce different social outcomes (see, e.g., Crenshaw 1989, 1991; Henne and Troshynski 2019; Potter 2013). Both social constructionism and intersectionality are central to many left liberal approaches, and both are engaged in deconstructing subjectivities into micro-categories, each of which are impacted in specific ways. According to the ultra-realists, however, without a unifying theory to critique capitalism, these left liberal approaches are doomed to remain as single-interest theories. Drawing on the work of the psychoanalyst Jacques Lacan (2006/1966; 1977) as it is filtered through Žižek (2006), Winlow and Hall (2013; see also Hall 2012) reject strain, labeling and subcultural theories, while acknowledging the influence of feminist and victimological research—but not their theoretical positions (see also Hall 2012). Winlow and Hall (2013) argue that these approaches (strain, labeling, subcultural theory) are isolated from each other ideologically and have fragmented criminology into multiple, and often competing, positions. Winlow and Hall’s problem with this is that the key source of harm is capitalism and hyper-consumerism. *This* should be the focus of change, but intersectional identity politics are distractions from this goal.

It is important to recall, here, that relative deprivation, as it has been developed outside of criminology, is considered to be an outcome of the comparisons of competing or contrasting reference groups. Only within pre-millennial left realism was relative deprivation linked directly to the anomie tradition of Merton and American subcultural theory, concomitant with the often uncritical acceptance of the American Dream (Webber 2007a). Consequently, the critique of left realism, labeling, and subcultural theory, for example, does not preclude the conceptual use of relative deprivation in its more nuanced, social psychological form by ultra-realists—or, indeed, any other perspective. The point is not that ultra-realists need to add another concept to their already rich soup of theorization, but that critics of relative deprivation need to be clear about what, exactly, they are critiquing. To claim that relative deprivation is just a subset of Mertonian strain theory is to miss the more complex approach taken by other scholars outside of criminology.

In addition, ultra-realists argue that ethnographic data can uncover the lived realities of social groupings, where lives are full of contradictions and counter-productive choices, and which render intersectional groups as constituted (falsely) by those who champion intersectionality. For example, the lived reality of people categorized as BAME or LGBQT will not be shared equally by everyone within in any given category. The harms of capitalism, exposed by ethnographic insight, are more powerful than the subcultures of identity politics. But viewing such lives through a lens of relative deprivation would reveal that no matter what economic system one lives under, one would still suffer deprivation because the creation of comparative reference groups is a social psychological reality regardless of politics. Consequently, for perspectives like ultra-realism that posit revolutionary change before progress can be made, comparative processes will continue to create intersections of various identities even after such monumental economic shifts have taken place.

There is no reason why relative deprivation, in its more elaborated and complexly rendered form, cannot be a key concept within ultra-realism. Its rejection is based on the argument that humans do not have a natural sense of justice, and perhaps also that it was shackled to left realism and that left realism was flawed as a pragmatic program of small administrative changes, rather than a wholesale undermining of the economic system that gave rise to such inequality. Relative deprivation, then, becomes a logically problematic idea because it suggests that there will always be inequalities regardless of the political economy. Powerful interest groups attack policies of financial redistribution not because they feel threatened that they might become poor, but because they have to give up something they regard as a *possession*, and as a key feature of one’s identity to make as much money as possible with few consequences and without consideration of others. Thus, for ultra-realists, relative deprivation might well be a useful explanation for crime, but it highlights the logical flaw in redistribution discourses: taking from the rich and distributing the resources to the poor does not guarantee peaceful equilibrium. Those who lose the most would see themselves as victims and seek to correct their loss. Regardless of whether or not ultra-realism might benefit from a renewed appreciation of relative deprivation as it has developed *beyond* left realism, the next part explores where relative deprivation has been employed to an analytically useful effect.

## Relative Deprivation in Social Psychology

So far, this article has focused on the absence and disappearance of a concept that was once central to a specific area of criminology. This part turns to how we might start to reimagine relative deprivation, in its elaborated form, as a key concept in criminology. As noted above, relative deprivation, as employed outside the field of criminology, is a more complex and more empirically grounded concept. Relative deprivation has been analyzed in many different contexts and countries, and has been explored by utilizing many different research methods (e.g., Smith et al. 2012). In Smith and colleagues’ (2012) review of studies of relative deprivation, the authors argue that *any* definition of relative deprivation must include a sense of deservingness. The person who feels deprived *ought* to feel that they are justifiably entitled to whatever it is that they are lacking. Indeed, as already argued, this sense of fair entitlement, following Rawls’ (1958) concept of “justice as fairness,” is central to Runciman’s account. Entitlement must be seen as socially just and value-free by an observer who does not know if one would benefit or lose from the outcome of the judgment. Someone who puts themselves in that same position should judge the sense of entitlement as being fair.

The concept of relative deprivation has been developed more fully within the social psychological approaches called “social identity theory” and “self-categorization theory” (Grant et al. 2015; Tajfel and Turner 1979; Turner 1985; Walker and Pettigrew 1984). These two theories share many concepts in common and are increasingly seen as inter-related (for a history of these approaches, see Hogg and Abrams 1999). For the purposes of this article, I will refer only to “social identity theory.” Psychologists working in the field of social identity theory have conducted many studies into relative deprivation (for a review, see Smith et al. 2012), but social identity theorists are not restricted to a focus on relative deprivation. Social identity theory is also concerned with cognition and motivation in laboratory conditions and asks why people perceive deprivation under certain conditions (Ellemers 2002). In this regard, relative deprivation has been inverted so that the processes through which individuals or groups arrive at a sense of well-being or injustice have become more important than the outcome of deprivation. So, for example, where the pre-millennial left realists were interested in the role of relative deprivation in causing crime, albeit with a “thin” account of the theory, social psychology concentrates on the way that individuals and groups create and compare in-groups with out-groups—in ways that *might then* result in anger or crime. In contrast, some in-groups and out-groups might engage in comparisons that do not result in obvious outcomes as when someone votes against their own best interests, or those who are content with their position in life when they are objectively worse off than other people from similar backgrounds. The traditional focus of pre-millennial left realism was that crime is considered the outcome of relative deprivation. In social psychology, deprivation might now be only *one possible outcome* of comparative processes, along with satisfaction or anger or, indeed, *any* other human emotion (Osborne and Sibley 2015; Pettigrew 2002; Smith and Pettigrew 2015). For pre-millennial left realism, relative deprivation leads to crime; for social psychology, comparative group processes result in various outcomes, such as anger, frustration, satisfaction—or relative deprivation. What happens next is another level of analytical inquiry, where crime, social movements and protest, or ambivalence and resignation, might be the result. In addition, the social psychological literature also deals with an issue that is rarely discussed in criminology—the temporal dimension. de la Sablonnière and colleagues (2015) have argued that psychological well-being is linked to an assessment of an individual or group’s assessment of their trajectory from the past to the future—what they refer to as “temporal relative deprivation” (see also Klandermans 2015; Sherif et al. 1961; Van den Bos et al. 2015). Again, this is a potentially useful approach for criminology, such as in youth transitions to adulthood.

The social psychological conception of relative deprivation, missing throughout left realist literature, is far more useful than the softly deterministic approach that criminology has come to understand, and now largely ignores. Pre-millennial left realism points to relative deprivation as a (straightforward) cause of crime. For Runciman, and the later elaborations in social psychology, relative deprivation is a far more complex set of processes. In other words, criminology is concerned with answering narrow questions of causative links which, in turn, has limited the way in which relative deprivation has been used in criminology. In contrast, by understanding the social comparative processes that lead to subjective outcomes, such as relative deprivation or relative satisfaction, we can begin to engage with and embrace Runciman’s consideration of Rawls’ (1958) concept of “social justice” and Runciman’s suggestion that it can remedy the negative effects of relative deprivation. Most importantly, and as argued above, a more capacious notion of relative deprivation is not incompatible with the more recent work of left realists and contemporary ultra-realists. Indeed, the latter is especially receptive to the subjective social positions that people and groups take.

## Relative Deprivation and the Precariat

In addition to social psychology, relative deprivation has been discussed in other areas of the social and political sciences. Several popular books have tackled questions of inequality and the precarious futures of neoliberal capitalism (e.g., Piketty 2014; Putnam 2000; Wilkinson and Pickett 2009). It is Guy Standing’s (2011) concept of the “precariat”—a portmanteau merging “precarious” and “proletarian”—that contains many overlaps with relative deprivation. According to Standing (2011: 33),

The precariat experiences the four A’s—anger, anomie, anxiety and alienation. The anger stems from frustration at the seemingly blocked avenues for advancing a meaningful life and from a sense of relative deprivation. Some would call that envy, but to be surrounded and constantly bombarded with the trappings of material success and the celebrity culture is bound to induce seething resentment.

Standing’s concept of the precariat raises the question of whether or not criminologists should utilize it instead of trying to resurrect an old idea like relative deprivation that has fallen out of fashion due to its link with a theoretical paradigm in criminology, left realism, which in its post-millennial form has ignored and neglected relative deprivation. There are, as yet, few academic discussions in criminology using the precariat as a central focus. Aside from a themed section of *Criminal Justice Matters* organized by Squires (2013) and a discussion in Valeria Vegh Weis’s (2017) book, *Marxism and Criminology,* the term, “precariat,” has been relatively absent. So, before moving on, it is necessary to see how similar the two terms are, wary as we should be of the complexities of this task given the way that relative deprivation has been used in sociological and psychological approaches.

As I have argued elsewhere (e.g., Webber 2007a), relative deprivation should be seen less as a *theory* than one possible outcome of comparative individual and group processes. Therefore, the outcome of a comparison, as exemplified in social psychological elaboration, can be relative deprivation, entitlement, gratification, or resentment, among many others (Feather 2015; LeBlanc et al. 2015). In terms of crime, there is no linear causative determinism: just because one feels resentment or deprivation, does not mean that one will commit an offense. It might, however, increase the possibility of that occurring. Standing’s work has certainly provided new considerations of precarious work in a world of increasing automation. But the nuanced research undertaken in social psychology on the effect of reference group comparisons on our sense of well-being remains a more robust and replicable method of understanding the effects of a political economy that has shifted toward the precarious. I would argue that this is possible only if it is aligned with a renewed focus on qualitative ethnographic methods. What this suggests is a synthesis of approaches that might start at the local level of lived experience through qualitative and ethnographic interviewing, but which also incorporates the social psychological methods of comparative attitudinal scales. What we have yet to establish is the role that a renewed and more robust approach to relative deprivation might still have as an explanation of crime because the idea is still missing in action in criminology.

In order to summarize this review thus far, Table 1 compares the perspectives outlined in this article, and serves as an extension of one that I presented in my first article on this subject (Webber 2007a: 105–106). It is intended as an overview and not as the final say on each approach.

**Table 1:** Summary of key theories in the link between relative deprivation and crime

Perspectives	Robert K. Merton (1938) and Social Structure and Anomie	W. G. Runciman (1966) and Relative Deprivation	Left realism (1984–1992)*	Guy Standing (2011-Present) and The Precariat	Ultra-Realism c.2012-Present (e.g., Hall 2012)
Key term(s)	Anomie: Although different to that used by Durkheim (see Box 1971). Merton accepted that crime rates show that crime is located mostly in the lower strata of society. This reversed the concept of anomie from Durkheim’s lack of regulation allowing the wealthy to reach for unattainable and dysfunctional goals, to one that argued that the route to the goal was blocked for certain groups.	Relative deprivation	Relative (economic) deprivation and political marginality; the Square of Crime; the “aetiological crisis.”	The Precariat/Precarity	Transcendental materialism (drawing on Hegel, Lacan and Žižek)
Concept of social order	Consensus; American Dream accepted widely	Plurality: society comprises a multitude of reference groups, although the saliency, or prominence, of any given factor can create consensus or conflict. For example, war can provide a unifying focus directed against an out-group. In addition, reference groups can shift and change depending upon the context.	Conflict: recognition of class-based categories demarcated by access to economic and political advantage.	Stratified into 7 groups with subsections: 1. Elite 2. The Salariat 3. The Proficians 4. The core, equivalent to the old working class, but lacking the shared class solidarity 1. The Precariat 2. The unemployed 3. The ill and outcasts	Malleable, indeterminate, and constituted by an intransitive realm, hidden from view, but effecting how things operate.
Problem	Strain determined, by anomie, leads to five modes of adaptation in response to blocked opportunity to reach the goal of success, “the American Dream.” 1. Conformity 2. Innovation 3. Ritualism 4. Retreatism 5. Rebellion Focused mainly on individual response to strain. Innovation main mode motivating criminal behavior.	Relative deprivation, defined as the excess of expectation over the opportunity to achieve it. This is contingent upon various factors, including comparisons at the level of group or individual. Relative deprivation is a value-neutral term; following Rawls’ notion of justice as fairness, feelings of envy or greed are not included; a third party must be able to recognize the social injustice of any sense of relative deprivation.	Crime can occur anywhere in the social structure, by the rich or the poor, and this is caused by relative economic deprivation and political marginality.	The Precariat is characterized by a lack of rights. This distinction is drawn through the use of the term, “denizen,” as an inferior position to that of a citizen. It is a class distinct from the working class and the proletariat, defined by the four “A’s”—anger, anomie, anxiety and alienation	Neoliberalism and consumerism are a central feature of life. This never-ending search for satisfaction through consumption—and where competition is the driving motivation for organizing every facet of life—creates the harms that can then be defined as crime. Everybody is complicit and aware that their activity causes harm—from the buying of “fast fashion,” to the exploitation behind cheap electronics—yet we continue to consume, and try to assuage our guilt through token gestures. All the while, the ultra-realists argue, the underlying causative economic and social problems remain largely unchallenged.
Response to problem	Adaptations which are empirically predictable. The goal of wealth and success—the “American Dream”—is accepted, and the routes to achieve it need to be available to all. Better education, better training, and better access to jobs, but redistribution of wealth and resources is not on the agenda.	Multitude of responses, can be in contrast to predicted outcome. People often do not regard themselves as deprived when they objectively are, and vice versa. Similarly, groups often vote against their own best interests.	Democratic policing or policing with the consent of the policed. Reducing relative economic deprivation, although how this might be achieved is relatively unelaborated.	Universal wage; raising collective awareness of those in precariat to forge a collective response.	The aim is to move the discussion of social injustice away from the proselytizing champagne socialism of a metropolitan middle-class commentariat, and toward those harmed most by rampant capitalism. To do this, we need to understand the deep structures of power and hidden forces acting on people. In this way, ultra-realism positions itself as an antidote to what it sees as the pragmatic, small-scale policy response of left realism.
Research method	Largely theoretical, elaborated empirically by later scholars, many of whom were doctoral students of Merton (e.g., A.K. Cohen; R. Cloward and W.G. Runciman).	1962 survey, designed specifically to key into relative deprivation.	Local victim surveys, such as the Islington Crime surveys (Jones et al. 1986).	Secondary data analysis of employment and demographic trends.	Ethnography to move beyond the neoliberal elite’s imposition of their own ideological practices. Generally accepting of Bhaskar’s methodology in critical realism. In particular, there is a call for a network of ethnographies of everyday life so that patterns can be seen across a region, country or class.
Types of crime explained by theory	Mainly acquisitive, although violence in pursuit of acquisition of profit can be included. Crime located mainly among the poor.	Runciman does not discuss crime, but the use of the idea in left realism is to explain any type of crime where there is a negative outcome from a comparison between in-group and out-group. Can explain crime at any level of the social structure, although left realism does not use Runciman’s approach in a systematic way.	Focusses mainly on intra-group working-class street crime.	The subheading of *The Precariat* is *The New Dangerous Class.* The danger is not crime, however. Indeed, crime is hardly mentioned in the book.	Returning to zemiology, ultra-realists are concerned with *all* forms of harm.
Inspiration for other scholars.	Albert Cohen (1955): Accepted Merton’s anomie, but looked at collective responses within working-class subcultures. Cloward and Ohlin (1960): Accepted anomie and collective response to strain, but added the concept of differential access to the illegitimate opportunity structure. Left realism utilized Merton’s anomie theory, calling it relative deprivation (Lea 1992). Relative deprivation *caused* crime. Robert Agnew’s General Strain Theory of Crime (1992).	Social psychological theories utilized Runciman’s concepts to explain individual, group and national levels of interaction. Social identity theory and the related self-categorization theory (Tajfel and Turner 1979) take as their focus reference groups, relative deprivation is one possible outcome of a negative self-concept. No discussion of crime in Runciman and few studies in social psychology. Relative deprivation explains a *tendency* toward crime.	Arguably the current crop of articles and books that explore left realism and refer to the earlier form (Lea 2002, 2016; Matthews 2016a, b) are elaborations because they share a common root, but drift from it in various ways. Jock Young also became a leading figure in cultural criminology (e.g., Ferrell et al. 2015). This represented a shift away from Young’s earlier position as a leading left realist, however, as he drew on phenomenological, anarchist and cultural studies approaches to crime and deviance.	Arguably, *The Precariat* is an elaboration of many other approaches. Its entire purpose is to elaborate prior research into class formation, labor and the economy in order to create its own elaboration, the precariat.	A few selected articles and books have used the ideas, but, as of yet, it has not been elaborated widely.
Criticism	Deterministic and uncritical of social order. Crimes of the powerful ignored, although the left realist elaboration argues that crime can occur anywhere in the social structure; street crime is still the main focus. Lacks a consideration of agency such that the foreground factors are ignored. For example, issues, such as gender, are neglected. If we accept the crime rates, as Merton did, then why are men more likely than women to commit crime?	Breadth of explanation too wide—potentially chaotic number of reference groups from which comparison can be made. Still leaves room for other theories to explain cause of crime.	The focus is on the poor and disadvantaged working class, despite the possibility for the theory’s more universal ambitions. Criticized for a male-centered approach. The reliance on relative deprivation is one that is confined to a Mertonian form of anomie theory, with little elaboration or connection to wider discussions beyond Merton. Runciman is hardly mentioned.	It can be argued that the idea is not new and that many people worked several jobs to make ends meet throughout history. The quest to describe and define a global movement renders the often unique economic histories of different nation states superfluous. It can also be argued that it undermines the quest to render working-class exploitation more visible by shifting the focus away from their struggles.	Sandra Walklate (2016) reviewed Hall and Winlow’s book (2015) and argued that it might be construed as a project in the making, rather than the finished article, if indeed any idea was ever finished. More profoundly, Walklate argues that it risks replacing one flawed criminology, for an equally problematic dogma.

*As noted above, it is questionable if the current wave of left realism is of the same form as its earlier incarnation and so this summary refers only to the pre-millennial version

## Bringing Back Relative Deprivation into the Crime Debate

This final part aims to reappraise the role of relative deprivation for criminological explanations of crime. So how might we imagine a return for the concept of relative deprivation into the analysis of crime? The original left realist project created theoretical positions against which it could be compared. One such approach to crime prevention was termed by the pre-millennial left realists as “new administrative criminology” (NAC) (see, e.g., Young 1997) to refer to the policy-oriented research of government departments. The term was loaded with a critical edge, and the “administrative” addition denoted the absence of theory (although see above the pointed criticism of left realists by Hall and Winlow (2015: 63) as “edgy administrators”). Encompassed within the NAC approach was Clarke and Cornish’s (1985) “rational choice theory” (RCT), which posits an account of the criminal as if they are a rational decision-maker using actuarial insight to make economic and risk-based decisions on whether or not to commit an offense. The overly calculating rational offender of RCT, *homo economicus*, has been criticized widely (e.g., Bouhana 2013; Hayward 2007). Rarely, however, has one of the key critiques of a simplified rational choice theory, albeit as it appears in economic theory, been applied to explain crime.

Kahneman and Tversky’s (1979) “prospect theory” aimed to show, through the application of psychological theory, where people make “thinking” errors. Cognitive biases, such as anchoring effects, cognitive dissonance, and confabulations, have been demonstrated widely in the psychological literature. This is referred to as “counterfactual thinking” (Kahneman and Miller 1986; Olson and Roese 2002). There is not the space to explore these here, but cognitive dissonance, for example, occurs where there is a contradiction between what one thinks and what one does. Smoking while knowing the risks of heart disease and lung cancer is a prime example. There are, to be sure, similar cognitive errors made when individuals make comparisons with others on any variable. The key question in relative deprivation is: why do we sometimes feel deprived, when we are not? Or, to put it another way, why are we satisfied with our lives, when we are objectively worse off than others? Relative deprivation is an idea that challenges the overly rational, calculating actor. This is because it contains within it the possibility that we can consider ourselves relatively happy despite being objectively poor, and vice versa. Similarly, Kahneman and Tversky’s (1979) prospect theory complicates *homo economicus* as suggested in RCT by positing psychological phenomena that undermine rationality because they work on the level of perception and attention, over which we have little rational control most of the time (see also Kahneman 2011; Tversky and Kahneman 1986).

Essentially, relative deprivation emerges as an outcome of comparative processes that reflect structural and agency positions, *and* contains some sense of what the new ultra-realists, following critical realists, would term *probabilistic tendencies* (Archer 1995, Bhaskar 1997/1975). This is a rejection of simple cause and effect, and an acceptance that there are underlying unconscious mechanisms at play that might be only vaguely uncovered by the tools of social science. This, as noted in Table 1, is the “intransitive realm,” although for Hall and Winlow (2015), following the lead of critical realism, the word *influence* is a better way to understand the effect of the intransitive realm. This realm incorporates ephemeral structures—forces and powers that are less amenable to anything other than superficial understanding through the methods of traditional empirical and positivistic social science.

There are things in the world that cannot be sensed or observed through traditional empirical inquiry, such as experiments. I would assert that relative deprivation sits at the intersection of the intransitive and transitive realms—at the junction of the unknowable and the knowable. The argument that relative deprivation is a useful tool to explain underlying structural forces and their inter-relationship with both agency and group responses is sound (even if some of the research cited from social psychology is more fully committed to a positivistic position that is rejected by both left realists and ultra-realists). This is exactly what the original formulation of relative deprivation, and its elaboration in social psychology, set out to accomplish. As I have argued above and elsewhere (e.g., Webber 2007a), relative deprivation should be seen not as a *cause* but as a *tendency* toward crime. There might be motivations to act in a certain way that are formed by layers of history. For example, for the “hard man,” his willingness to use violence might be unconscious and thus not discoverable in interviews or surveys. Relative deprivation *can* explain underlying structural forces and their inter-relationship with both agency and group responses. This is evidenced by a wealth of research from outside of criminology (e.g., Pettigrew 2002; Smith and Pettigrew 2015; Smith et al. 2012). In other words, relative deprivation occupies the liminal space at the intersection of objective factors and subjective experience. With its elaboration from within psychology, we can see how it allows for an appreciation of the often irrational, counter-intuitive thinking that characterizes much criminal motivation—and it also suggests routes to understanding tendencies that raise the possibility of crime in, for example, intergroup tensions, such as when race or immigration becomes salient (such as after the British vote to leave the European Union in 2016).

In sum, relative deprivation is not a panacea for criminology, and I do not claim that the social psychological research on reference groups is the answer to deep-seated criminological conundrums. Instead, I wish to assert that it is still a good idea to “think with”—an idea that seems to have disappeared not through any lack of analytical robustness it might bring. Effectively, relative deprivation has been neglected because it has fallen out of fashion and so has not made the jump to the post-millennial form of left realism, except for a few exceptions, and even then, and again, not in the elaborated sense that I have promoted in this article. Relative deprivation, as a concept, is now coupled with the tendency toward disdain that sociological criminology has toward psychological ideas, and so the idea has become outdated (Webber 2020). But, as I have maintained in this article, relative deprivation, in its more nuanced form, is more important than ever. We have entered a period of rampant consumerism coupled with a network-enabled new administrative criminology, and with security services rapidly collaborating with, and supporting, universities. Crime science has become focused on prevention through design. But now, “designing out” crime is no longer limited to target hardening or the defensive qualities of a building or the location of closed-circuit television (CCTV), which were once the focus of (administrative) criminological investigations. It is now oriented toward algorithms and better designed software and Big Data analytics that have become a central concern of, mainly, computer science departments around the world. Consequently, there is a need for a qualitative or ethnographic shift toward prevention through social change—something that criminology *can* pursue. If we take cybercrime as an example, it is still early in our understanding of why young people might shift from using technology for games and a chat to buying and selling stolen credit card data on carding forums. In addition, when an explanation is presented to explain young people’s behavior, it invariably entails RCT (Webber and Yip 2019). The robust analytical constructs of comparative reference group theory that underpins the elaborated form of relative deprivation are an untapped resource in criminology.

One lesson to take from Standing’s (2011) precariat thesis is that old divisions based on class have changed. Many people who might once have found themselves categorized as middle class can have occupations that are precarious. In the election in the UK in 2019, Boris Johnson’s Conservative Party returned to power with an increased majority by appealing to working-class voters in previously strong Labor seats. Similarly, in the US in 2016, Donald J. Trump also argued that he was the candidate for the blue-collar workers and won the presidency, albeit without winning the majority of votes. Through the lens of relative deprivation, or more specifically, the shifting reference group comparisons and the concomitant behavioral outcomes, we can find more ways to understand the complexities of a hyper-networked, consumerist society. There are shifts taking place that might presage a positive route through what, for many progressive thinkers, is only a depressing resurrection of ideas that were once thought slain. The rise of white supremacy and the normalization of sexism, racism and anti-LGBTQ people has been challenged by #MeToo, Black Lives Matter protests, and LGBTQ rights. Yet, even so, reference group distinctions drawn along binary political and ideological positions have become more, not less, polarized and poisonous. There is an urgent need to identify a shared narrative, at the national, international, or local and individual level, that can take the place of divisive points of contrast. Here, the greatest self-inflicted harm might provide the impetus. The threat of ecological destruction has shifted from the bipolarity of accepters and deniers to, increasingly, a grim awakening that economic, industrial and social systems need to change radically or risk climate-linked mass migration, poverty and precarity. Such a narrative might suggest an alternative solution that can hold a fractured citizenry in balance, rather than opposition. A shared goal on a global scale that requires cooperation might be the best (worst) option.

One positive outcome of the global COVID-19 pandemic is that entire countries allowed their freedoms to be restricted for the common good of preventing the spread of a deadly disease. As I write this in the social isolation required of British citizens, the pandemic is still dominating and destroying the lives of people throughout the whole world. Global cooperation has now been accompanied by the protests of Black Lives Matter. The shifts in attitudes, as previously well-defined reference groups have fractured, have yet to settle into an identifiable whole. It may be that the shifts become permanent and that the attitudes to collective living, either in terms of public health, race or sexuality, become more collaborative and caring. Alternatively, we might witness even more hardened oppositional groups and clearer lines of demarcation—or, worse, newly constituted oppositional groups, which could mean more harmful rhetoric, more crime and more terrorism. Relative deprivation—and the underlying theories of comparative reference groups, human behavior, and stereotyping—can help us understand these monumental changes. An appeal to social justice, if loud enough, can shift trenchantly held opinions.

## Footnotes

1 In the interests of gender neutrality, I have elected to use “they,” “their,” and “themselves” rather than “he” or “she” (or “he/she”), “his” or “her” (or “his/her”), and “himself” or “herself” (or “himself/herself”) in this article.
